# Postoperative adjuvant chemotherapy versus chemoradiotherapy for node-positive esophageal squamous cell carcinoma: a propensity score-matched analysis

**DOI:** 10.1186/s13014-020-01557-9

**Published:** 2020-05-24

**Authors:** Qifeng Wang, Jinyi Lang, Tao Li, Lin Peng, Wei Dai, Yinchun Jiang, Tianpeng Xie, Qiang Fang, Yi Wang, Lei Wu, Bangrong Cao, Yongtao Han

**Affiliations:** 1 Department of Radiation Oncology,Sichuan Cancer Hospital & Institution, Sichuan Cancer Center, University of Electronic Science and Technology of China, Radiation Oncology Key Laboratory of Sichuan Province, Chengdu, 610041 China; 2 Department of Medical Oncology,Sichuan Cancer Hospital & Institution, Sichuan Cancer Center, University of Electronic Science and Technology of China, Radiation Oncology Key Laboratory of Sichuan Province, Chengdu, 610041 China; 3 Department of Thoracic Surgery, Sichuan Cancer Hospital & Institution, Sichuan Cancer Center, School of Medicine, University of Electronic Science and Technology of China, Radiation Oncology Key Laboratory of Sichuan Province, Chengdu, 610041 China

**Keywords:** Adjuvant therapy, Esophagectomy, Esophageal cancer, Chemotherapy, Chemoradiotherapy

## Abstract

**Background and purpose:**

After esophagectomy, adjuvant chemotherapy (S + CT) and adjuvant chemoradiotherapy (S + CRT) can improve survival in patients with node-positive resectable esophageal cancer. However, we are not aware of any studies that directly compared these adjuvant treatments. This study aimed to compare S + CT and S + CRT for patients with esophageal cancer.

**Materials and methods:**

We retrospectively identified patients with node-positive esophageal squamous cell carcinoma who underwent S + CT or S + CRT at Sichuan Cancer Hospital during 2008–2017. The patients’ characteristics were compared, as well as their overall survival (OS) and disease-free survival (DFS) outcomes. Propensity score matching was used to create balanced patient groups according to adjuvant treatment, and a Cox proportional hazards model was used to identify factors that predicted the survival outcomes.

**Results:**

The 859 eligible patients underwent S + CRT (250 patients, 29.1%) or S + CT (609 patients, 70.9%). After propensity score matching (247 patients per group), the 5-year OS rates were 41.8% for S + CRT and 26.8% for S + CT (*p* = 0.028), and the 5-year DFS rates were 37.2% for S + CRT and 25.5% for S + CT (*p* = 0.012). Multivariate Cox regression analysis of the matched samples revealed that, relative to the S + CT group, the S + CRT group had better OS (hazard ratio: 0.71, 95% CI: 0.56–0.91; *p* = 0.006) and DFS (hazard ratio: 0.70, 95% CI: 0.56–0.88; *p* = 0.002).

**Conclusion:**

Among patients with node-positive resectable esophageal squamous cell carcinoma, S + CRT was associated with better OS than S + CT. A multicenter randomized clinical trial is warranted to confirm these findings.

## Introduction

Esophageal cancer is associated with a poor prognosis and substantial mortality rate [1, 2]. Although esophagectomy is the main treatment option for patients with localized advanced esophageal cancer, the 5-year overall survival (OS) rate remains < 20% after surgery alone [3–7]. Furthermore, patients with pathologic lymph node LN metastasis have a significantly lower survival rate than patients without LN metastasis [4, 5, 8, 9]. Several studies have indicated that, relative to surgery alone, multimodality treatment significantly improves OS among patients with locally advanced esophageal cancer [5, 8, 10–13]. The main additional treatment for locally advanced esophageal squamous cell carcinoma and adenocarcinoma is neoadjuvant chemoradiotherapy (NCRT), based on the results from the CROSS study [11] and the NEOCRTEC5010 study [10]. Recently some researchers reported persistent pathologic LN metastasis with or without NCRT is a strong poor prognostic factor in ESCC [14, 15]. Therefore, an effective preoperative or postoperative treatment (e.g., radiotherapy and/or chemotherapy) is needed to improve outcomes, especially for patients with pathologically node-positive (pN+) esophageal cancer. Postoperative chemotherapy and/or radiotherapy provide significantly better long-term survival, relative to surgery alone, for patients with pN+ esophageal cancer [3, 5, 7, 13]. However, to the best of our knowledge, no studies have directly compared S + CT and S + CRT for pN+ esophageal squamous cell carcinoma (ESCC). Therefore, this retrospective study examined whether S + CT or S + CRT were associated with improved survival among patients with pN+ ESCC.

## Patients and methods

### Patient selection

**Fig. 1:**
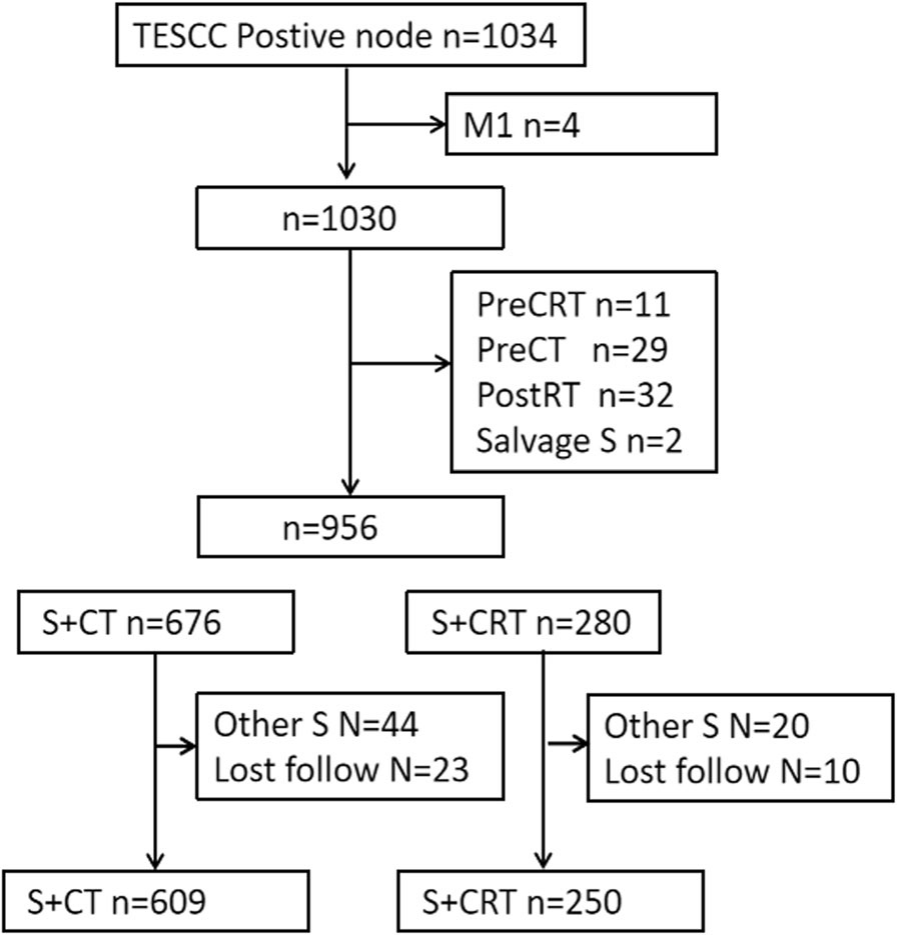
Study flowchart. ESCC: esophageal squamous cell carcinoma; PreCRT: preoperative chemoradiotherapy; PreCT: preoperative chemotherapy; PostRT: postoperative radiotherapy; S: surgery; S + CT: postoperative chemotherapy; S + CRT: postoperative chemoradiotherapy

Between January 2008 and December 2017, we retrospectively identified 1034 patients who underwent esophagectomy with curative intent (Fig 1). The eligibility criteria were: (1) histologically proven thoracic ESCC; (2) R0 and R1-2 resection; (3) standard McKeown esophagectomy or Ivor-Lewis esophagectomy; (4) pathological classification of any size tumor (T1–T4), regional lymph node metastasis (N1–3), and no distant metastases (pathological stage IIb, III, or IVa); (5) age of ≥18 years and Karnofsky performance status (KPS) of ≥80; (6) adequate bone marrow, renal, and hepatic functions; and (7) underwent postoperative chemotherapy or chemoradiotherapy. The exclusion criteria were cervical esophageal tumors, adenocarcinoma, and small cell carcinoma. The study’s retrospective protocol was approved by the appropriate institutional review board.

### Surgery

All patients received intravenous and inhalation-based general anesthesia. The surgical techniques were standard McKeown esophagectomy (*n* = 343) or Ivor-Lewis esophagectomy (*n* = 516). The surgical approaches were minimally invasive esophagectomy (*n* = 319) and open esophagectomy (*n* = 540). The surgical specimen was retrospectively restaged based on the 8th edition of the TNM classification for esophageal cancer [5].

### Chemotherapy

The S + CT group included 609 patients and the S + CRT group included 250 patients. Cisplatin-based regimens were used for 196 patients in the S + CT group and 124 patients in the S + CRT group. Nedaplatin-based regimens were used for 239 patients in the S + CT group and 32 patients in the S + CRT group. Oxaliplatin-based regimens were used for 131 patients in the S + CT group and 50 patients in the S + CRT group. Carboplatin-based regimens were used for 13 patients in the S + CT group and 1 patient in the S + CRT group. In addition, Gimeracil and Oteracil Porassium Capsules (S1) treatment was provided for 30 patients in the S + CT group and 43 patients in the S + CRT group. In the S + CRT group, 116 patients received concurrent chemoradiation with sequential chemotherapy and 134 patients received concurrent chemoradiation without sequential chemotherapy.

### Radiotherapy

Radiotherapy was started at 4–10 weeks after surgery. Computed tomography was used to identify anatomical landmarks and delineate the mediastinal lymph node stations. The clinical target volume was defined as both the tumor bed and high-risk lymphatic drainage areas. Postoperative radiotherapy was avoided for patients with a gastric tube located at the primary esophageal bed. Any anastomosis was included in the clinical target volume for patients with upper thoracic tumors and patients who had an insufficient proximal margin (< 3 cm). The radiotherapy involved a total dose of 50–54 Gy delivered to 95% of the planning target volume in 25–30 fractions (5 fractions/week for 5–6 weeks).

### Outcomes

All patients were followed every 3–6 months for the first 2 years after treatment, every 6–12 months for the following 3 years, and then annually thereafter. The follow-up evaluations included computed tomography scans of the neck, chest, and upper abdomen; ultrasonographic examination of the neck and upper abdomen; nuclear bone scanning; and blood routine test and biochemical tests. Esophagogastroscopy, positron emission tomography-computed tomography, and fine-needle aspiration cytology were performed when necessary. Recurrence was defined as locoregional recurrence (LRR, recurrence in the supraclavicular, mediastinal, and peritoneal regions) or distant metastasis (any other form of recurrence), and the relapse was confirmed via computed tomography, magnetic resonance imaging, or endoscopic examination of the corresponding site. Cytology or histology was performed if necessary. Multiple relapses detected within 1 month were considered synchronous.

The OS interval was calculated from the date of surgery until the date of death or the last follow-up, with surviving patients being censored at the last date of contact. The disease-free survival (DFS) interval was the length of time after primary treatment for a cancer ends that the patient survives without any signs or symptoms, with surviving patients being censored at the last date without any evidence of relapse.

### Statistical analysis

The groups’ OS and DFS values were compared using the Kaplan-Meier method and the unstratified log-rank test. A Cox regression model with stepwise selection was used for the multivariate analyses, and the results were reported as hazard ratios (HRs) with 95% confidence intervals (CIs). To further adjust for unbalanced covariates, we performed propensity score matching to create two comparable groups of patients who underwent surgery plus adjuvant chemotherapy (the S + CT group) or surgery plus adjuvant chemoradiotherapy (the S + CRT group). The patients’ propensity scores were estimated using a logit model that included age, sex, KPS, weight loss, tumor length, tumor location, pathological grade, lymphovascular invasion, nerve invasion, number of resected nodes, and TNM stage. Nearest neighbor matching (1:1) was performed without replacement based on a prespecified caliper width to match patients in the S + CT and S + CRT groups. Differences were considered statistically significant at *p*-values of < 0.05.

## Results

### Patient characteristics

Between January 2008 and December 2017, we retrospectively identified 1787 consecutive patients with pN+ ESCC. Based on the inclusion and exclusion criteria, we included 859 patients who underwent either S + CT (609 patients) or S + CRT (250 patients). The patients’ clinical characteristics are shown in Table 1. The two groups had similar clinical and pathological characteristics (e.g., age, sex, tumor status, histology differentiation, vascular invasion, nerve invasion, and node dissection). However, relative to the S + CT group, the S + CRT group had significant better performance status (*p* = 0.001), more upper site locations (*p* < 0.001), more R1–2 margins (*p* = 0.002), and more two-field lymph node dissections (*p* < 0.001). The S + CT group was more likely to have pT3 status than the S + CRT group (*p* = 0.021). After the propensity score matching, the two matched groups had similar clinical and pathological characteristics (Table 1).

**Table 1:** Clinicopathological characteristics of the patients before and after propensity score matching

		Before PSM				After PSM		
	All ***n*** = 859	S + CT ***n*** = 609 (%)	S + CRT ***n*** = 250 (%)	P-value	All ***n*** = 494	S + CT ***n*** = 247(%)	S + CRT n = 247 (%)	P-value
***Age, years***				**0.301**				**1.000**
**≤65**	**674(78.46)**	**484(79.5)**	**190(76)**		**377(76.32)**	**189(76.5)**	**188(76.1)**	
**> 65**	**185(21.54)**	**125(20.5)**	**60(24)**		**117(23.68)**	**58(23.5)**	**59(23.9)**	
***Sex***				**0.580**				**0.215**
**Male**	**735(85.56)**	**518(85.1)**	**217(86.8)**		**417(84.41)**	**203(82.2)**	**214(86.6)**	
**Female**	**124(14.44)**	**91(14.9)**	**33(13.2)**		**77(15.59)**	**44(17.8)**	**33(13.4)**	
***KPS score***				**0.001**				**0.786**
**90-100**	**399(46.45)**	**260(42.7)**	**139(55.6)**		**278(56.28)**	**141(57.1)**	**137(55.5)**	
**70-80**	**460(53.55)**	**349(57.3)**	**111(44.4)**		**216(43.72)**	**106(42.9)**	**110(44.5)**	
**Surgery type**				**0.010**				**0.279**
**Open**	**540(62.86)**	**400(65.7)**	**140(56)**		**265(53.64)**	**126(51)**	**139(56.3)**	
**MIE**	**319(37.14)**	**209(34.3)**	**110(44)**		**229(46.36)**	**121(49)**	**108(43.7)**	
**lymph node dissection**				**< 0.001**				**0.917**
**two fields**	**516(60.07)**	**326(53.5)**	**190(76)**		**372(75.3)**	**185(74.9)**	**187(75.7)**	
**three fields**	**343(39.93)**	**283(46.5)**	**60(24)**		**122(24.7)**	**62(25.1)**	**60(24.3)**	
***Pathological differentiation***				**0.305**				**0.671**
**Well (G1)**	**111(12.92)**	**76(12.5)**	**35(14)**		**73(14.78)**	**39(15.8)**	**34(13.8)**	
**Moderate (G2)**	**366(42.61)**	**252(41.4)**	**114(45.6)**		**215(43.52)**	**103(41.7)**	**112(45.3)**	
**Poor or undifferentiated (G3–4)**	**382(44.47)**	**281(46.1)**	**101(40.4)**		**206(41.7)**	**105(42.5)**	**101(40.9)**	
***Location***				**< 0.001**				**0.449**
**Upper site**	**166(19.32)**	**92(15.1)**	**74(29.6)**		**134(27.13)**	**62(25.1)**	**72(29.1)**	
**Middle site**	**456(53.08)**	**323(53)**	**133(53.2)**		**265(53.64)**	**133(53.8)**	**132(53.4)**	
**Lower site**	**237(27.59)**	**194(31.9)**	**43(17.2)**		**95(19.23)**	**52(21.1)**	**43(17.4)**	
**Resection**				**0.002**				**0.430**
**R0**	**803(93.48)**	**580(95.2)**	**223(89.2)**		**450(91.09)**	**228(92.3)**	**222(89.9)**	
**R1-2**	**56(6.52)**	**29(4.8)**	**27(10.8)**		**44(8.91)**	**19(7.7)**	**25(10.1)**	
**Varscular Invasion**				**0.210**				**0.397**
**Yes**	**647(75.32)**	**451(74.1)**	**196(78.4)**		**377(76.32)**	**184(74.5)**	**193(78.1)**	
**No**	**212(24.68)**	**158(25.9)**	**54(21.6)**		**117(23.68)**	**63(25.5)**	**54(21.9)**	
***Nerve invasion***				**1.000**				**0.391**
**Yes**	**677(78.81)**	**480(78.8)**	**197(78.8)**		**381(77.13)**	**186(75.3)**	**195(78.9)**	
**No**	**182(21.19)**	**129(21.2)**	**53(21.2)**		**113(22.87)**	**61(24.7)**	**52(21.1)**	
***Dissection nodes***				**0.394**				**0.652**
**≤20**	**406(47.26)**	**294(48.3)**	**112(44.8)**		**226(45.75)**	**116(47)**	**110(44.5)**	
**> 20**	**453(52.74)**	**315(51.7)**	**138(55.2)**		**268(54.25)**	**131(53)**	**137(55.5)**	
***Pathological T status***				**< 0.001**				**0.130**
**T1**	**42(4.89)**	**30(4.9)**	**12(4.8)**		**18(3.64)**	**6(2.4)**	**12(4.9)**	
**T2**	**148(17.23)**	**110(18.1)**	**38(15.2)**		**74(14.98)**	**36(14.6)**	**38(15.4)**	
**T3**	**573(66.71)**	**419(68.8)**	**154(61.6)**		**329(66.6)**	**175(70.9)**	**154(62.3)**	
**T4a**	**87(10.13)**	**49(8)**	**38(15.2)**		**67(13.56)**	**29(11.7)**	**38(15.4)**	
**T4b**	**9(1.05)**	**1(0.2)**	**8(3.2)**		**6(1.21)**	**1(0.4)**	**5(2)**	
***Pathological N status***				**0.057**				**0.327**
**N1**	**455(52.97)**	**320(52.5)**	**135(54)**		**281(56.88)**	**148(59.9)**	**133(53.8)**	
**N2**	**270(31.43)**	**183(30)**	**87(34.8)**		**157(31.78)**	**71(28.7)**	**86(34.8)**	
**N3**	**134(15.6)**	**106(17.4)**	**28(11.2)**		**56(11.34)**	**28(11.3)**	**28(11.3)**	
***Pathological TNM stage***				**0.896**				**0.629**
**IIB**	**28(3.26)**	**20(3.3)**	**8(3.2)**		**13(2.63)**	**5(2)**	**8(3.2)**	
**IIIA**	**97(11.29)**	**72(11.8)**	**25(10)**		**52(10.53)**	**27(10.9)**	**25(10.1)**	
**IIIB**	**562(65.42)**	**396(65)**	**166(66.4)**		**341(69.03)**	**175(70.9)**	**166(67.2)**	
**IVA**	**172(20.02)**	**121(19.9)**	**51(20.4)**		**88(17.81)**	**40(16.2)**	**48(19.4)**	

Note: *AJCC* American Joint Committee on Cancer, *S* Surgery alone, *S + CT* Postoperative chemotherapy, *S + CRT* postoperative chemoradiotherapy, *OS* Overall survival, *DFS* Disease-free survival, *HR* Hazard ratio, *CI* Confident interval, *KPS* Karnofsky Performance Status, *MIE* Minimally invasive esophagectomy

### Survival

**Fig. 2:**
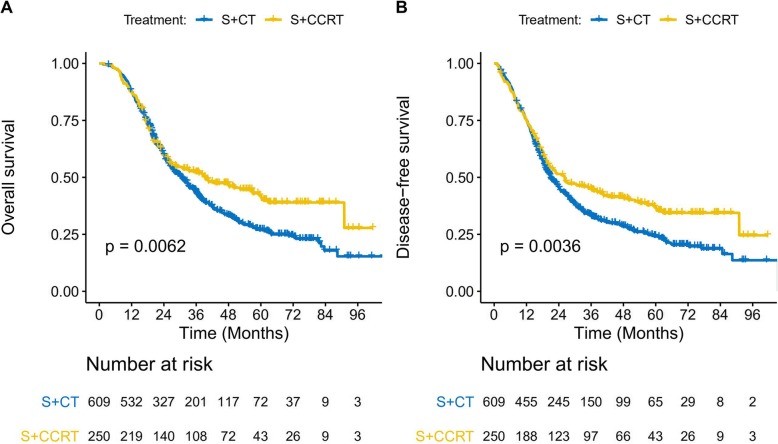
Overall survival (**a**) and disease-free survival (**b**) among the entire cohort. NO.: number; S: surgery, S + CT: postoperative chemotherapy; S + CRT: postoperative chemoradiotherapy

The median follow-up for all patients was 42.5 months (range: 3–116 months), with a 3-year OS rate of 46.2% and a 5-year OS rate of 31.7% (median OS: 31.7 months). The 3-year DFS rate was 37.0% and the 5-year DFS rate was 28.1% (median DFS: 21.9 months). Relative to the S + CT group (3-year OS: 43.5%, 5-year OS: 27.0%, median OS: 30.4 months), the S + CRT group had significantly better OS outcomes (3-year OS: 52.2%, 5-year OS: 42.0%, median OS: 39.3 months; *p* = 0.006) (Fig. 2a). Relative to the S + CT group (3-year DFS: 33.4%, 5-year DFS: 24.3%, median DFS: 21 months), the S + CRT group had significantly longer DFS outcomes (3-year DFS: 44.9%, 5-year DFS: 37.1%, median DFS: 26.2 months; *p* = 0.004) (Fig. 2b).

### Outcomes in the matched groups

**Fig. 3:**
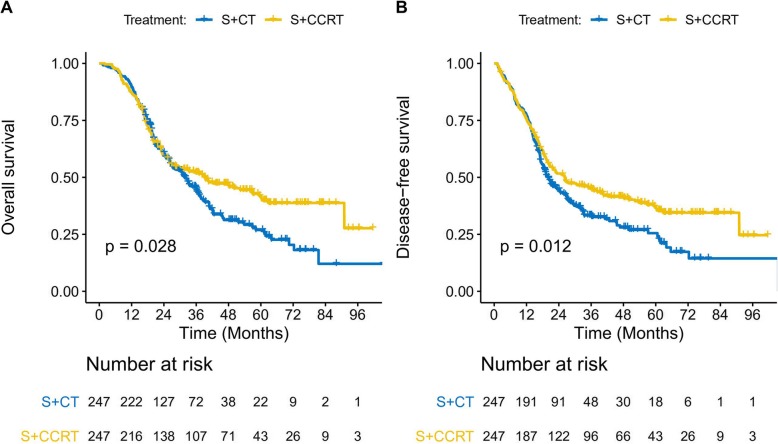
Overall survival (**a**) and disease-free survival (**b**) among the matched cases. NO.: number, S + CT: postoperative chemotherapy; S + CRT: postoperative chemoradiotherapy

When we considered the propensity score-matched groups, the S + CT group had poorer OS outcomes (3-year OS: 45.3%, 5-year OS: 26.8%, median OS: 32.1 months) than the S + CRT group (3-year OS: 52.0%, 5-year OS: 41.8%, median OS: 39.3 months; *p* = 0.023) (Fig. 3a). In addition, the S + CT group had poorer DFS outcomes (3-year DFS: 33.3%, 5-year DFS: 25.5%, median DFS: 2.1 months) than the S + CRT group (3-year DFS: 45.1%, 5-year DFS: 37.1%, median DFS: 26.2 months; *p* = 0.012) (Fig. 3b).

### Univariate and multivariate analyses of the matched groups

Univariate analyses revealed that the survival outcomes were associated with the KPS score, surgical technique, surgical margin, vascular invasion, nerve invasion, and postoperative pathological T/N status (Supplementary Table 1). When we considered the propensity score-matched groups, the multivariate Cox regression analyses confirmed that S + CRT was independently associated with better OS, relative to S + CT (HR: 0.71, 95% CI: 0.56–0.91; *p* = 0.006) (Table 2). In addition, OS was independently predicted by KPS score, number of dissected nodes, surgical margin, vascular invasion, and pathological T/N status. Furthermore, in the propensity score-matched groups, S + CRT was associated with better DFS, relative to S + CT (HR: 0.70, 95% CI: 0.56–0.88; *p* = 0.002). The other independent predictors of DFS were KPS score and pathological T/N status.

**Table 2:** Multivariate analysis of factors influencing overall survival and disease-free survival in the matched groups(Multivariate Cox regression for both *P* < 0.1 for OS and DFS in Univariate analysis)

		Overall survival		Disease free survival	
		HR(95%CI)	***P*** value	HR(95%CI)	***P*** value
**KPS**	**90-100**	**1**		**1**	
	**70-80**	**1.35(1.06-1.71)**	**0.014**	**1.3(1.03-1.63)**	**0.025**
**Operation type**	**Open**	**1**		**1**	
	**mini**	**0.86(0.67-1.11)**	**0.255**	**0.8(0.63-1.02)**	**0.070**
**Margin**	**R0**	**1**		**1**	
	**R1-2**	**1.53(1.05-2.22)**	**0.028**	**1.22(0.84-1.79)**	**0.294**
**Varscular Invasion**	**No**	**1**		**1**	
	**Yes**	**1.34(1.02-1.78)**	**0.037**	**1.18(0.91-1.54)**	**0.220**
**Neuro Invasion**	**No**	**1**		**1**	
	**Yes**	**1.02(0.76-1.36)**	**0.903**	**1.22(0.93-1.61)**	**0.153**
**Path T stage**	**T1**	**1**		**1**	
	**T2**	**2.22(0.78-6.31)**	**0.134**	**1.48(0.66-3.33)**	**0.347**
	**T3**	**2.53(0.93-6.86)**	**0.069**	**1.66(0.78-3.56)**	**0.192**
	**T4a**	**3.79(1.34-10.71)**	**0.012**	**2.19(0.97-4.93)**	**0.059**
	**T4b**	**22.24(6.04-81.86)**	**0.000**	**8.12(2.61-25.3)**	**0.000**
**Path N stage**	**N1**				
	**N2**	**2.05(1.58-2.65)**	**0.000**	**1.85(1.44-2.37)**	**0.000**
	**N3**	**2.65(1.86-3.76)**	**0.000**	**2.45(1.74-3.45)**	**0.000**
**Adjuvant Therapy**	**S + CT**				
	**S + CRT**	**0.71(0.56-0.91)**	**0.006**	**0.7(0.56-0.88)**	**0.002**

### Subgroup analyses in the matched groups

For patients with KPS scores of 90-100, the S + CRT group had similar 5-year rates of OS and DFS, relative to the S + CT group. For patient with KPS scores of 70–80, the S + CRT group had significantly better 5-year rates of OS (39.1% vs. 17.8%, *p* = 0.011) and DFS (31.9% vs. 13.7%, *p* = 0.038) (Supporting Figure 1). For patients with pT1–2 status, the S + CRT group had similar 5-year rates of OS and DFS, relative to the S + CT group. For patients with pT3–4 status, the S + CRT group had significantly better 5-year rates of OS (39.6% vs. 23.9%, *p* = 0.043) and DFS (35.2% vs. 23.2%, *p* = 0.016) (Supporting Figure 2D). For patients with pN2–3 status, the S + CRT group had similar 5-year rates of OS and DFS, relative to the S + CT group. For patient with pN1 status, the S + CRT group had significantly better 5-year rates of OS (57.3% vs. 36.4%, *p* = 0.007) and DFS (52.1% vs. 36.5%, *p* = 0.007) (Supporting Figure 3).

### Patterns of failure in the matched groups

Based on the findings at the last follow-up, the local-regional recurrence rate was significantly lower in the S + CRT group than in the S + CT group (27.1% [67/247] vs. 34.8% [86/247], *p* = 0.023). In the S + CRT group, the recurrences involved the mediastinal lymph nodes (16.2%,[40/247]), the supraclavicular lymph nodes (8.1%,[20/247]), and the abdominal lymph nodes (1.6%,[4/247]), with 7 cases (2.8%,[7/247])involving anastomotic recurrence (1 case related to an esophageal fistula). In the S + CT group, the recurrences involved the mediastinal lymph nodes (22.7%,[56/247]), the supraclavicular lymph nodes (6.5%,[16/247]), and the abdominal lymph nodes (4.9%,[12/247]), with 2 cases involving anastomotic recurrence (0.8%,[2/247]). The two groups had similar rates of distant metastasis (S + CRT: 45/247 [18.2%], S + CT: 47/247 [19.0%]; *p* = 0.779), which commonly involved the lungs (*n* = 37,[20/247]), liver (*n* = 25,[20/247]), bones (*n* = 21,[20/247]) and other organs (*n* = 9).

## Discussion

The current National Comprehensive Cancer Network guidelines [17] recommend no additional treatment for squamous esophageal cancer unless the surgical margins are positive, although even patients with complete resection have a poor prognosis. The present study evaluated patients with pN+ ESCC and found that S + CRT was associated with a survival advantage in terms of OS and DFS, relative to S + CT. Furthermore, this advantage was still observed when we compared the propensity score-matched groups. Moreover, the independent predictors of OS and DFS were S + CRT, KPS score, and pathological T/N status. Finally, S + CRT was associated with a significantly lower LRR (relative to S + CT), although S + CRT did not appear to affect the rate of distant metastasis.

Surgical techniques for esophageal cancer have improved dramatically over the last decade, from two-field dissection to three-field dissection and from open surgery to video-assisted thoracoscopic minimally invasive surgery [16–18]. These advances have led to improvements in survival. However, local recurrence remains the main cause of treatment failure among patients with locally advanced tumors (41.5-49% of cases) [17, 19, 20]. A few studies reported that additional preoperative and postoperative therapy is necessary for patients with pN+ disease [3, 4, 8, 22–24]. Hsu et al. also reported that, among pN+ patients, S + CRT provided better outcomes than surgery alone in terms of 3-year OS (45.8% vs. 14.1%, *p* < 0.001) and 3-year DFS (24.1% vs. 11.5%, *p* = 0.0002) [25]. Furthermore, Lee et al. reported that S + CT was associated with a significantly better 3-year DFS rate than surgery alone (47.6% vs. 35.6%, *p* = 0.049) [4]. Thus, these studies have consistently indicated that adding postoperative chemotherapy and/or radiotherapy was able to improve OS among patients with pN+ disease, relative to surgery alone [3,4,6,8,16,25]. However, no studies have directly attempted to determine whether S + CT or S + CRT is preferable in this setting, and the present study aimed to address this issue. The results from before and after the propensity score-matching indicate that S + CRT provided better long-term OS and DFS, relative to S + CT.

Interestingly, Bedard et al. [21] have reported that S + CRT can cause biological changes in the tumor and reduce the possibility of subclinical or local recurrence. For patients underwent NCRT in CROSS study [11], the local regional recurrence have decrease significant (Anastomosis: 2.8% vs 8.7%, *P* = 0.008, Mediastinum: 7.0% vs 20.5%, P<0.001). Only 9 patients have recurrence in RT field. The results showed that local radiotherapy plus surgery could significantly reduce the LRR, and the LRR in the irradiated field was even lower. The present study also revealed that S + CRT was associated with a significantly lower LRR, especially for mediastinal recurrences, relative to S + CT. Thus, it is possible that ESCC may be sensitive to CRT, which might explain why S + CRT was more effective at improving the long-term OS and DFS outcomes. Furthermore, many studies have indicated that preoperative chemoradiotherapy significantly increases the pCR, relative to preoperative chemotherapy [10–12, 22, 23]. Moreover, improvements in radiotherapy techniques have helped improve OS outcomes for locally advanced esophageal cancer, and improved surgical techniques have resulted in smaller postoperative radiotherapy targets [9, 24]. Unfortunately, a radiation field limited to the mediastinal region is associated with a high supraclavicular and abdominal recurrence rate [13]. Thus, more precise treatments need to balance efficacy and treatment-related side effects, which suggest that further studies are needed to clarify the most appropriate chemotherapy regimens and radiation doses and target volumes.

The present study has several limitations. First, the retrospective data collection over a 10-year period is prone to bias and confounding, although we attempted to minimize the effects by using propensity score matching to create two relatively comparable groups. For example, patients with KPS 90-100 can benefit from the S + CRT. Second, there have been various improvements in radiotherapy, chemotherapy, and surgical techniques during the 10-year period, which might have influenced our findings. Third, we only considered patients who were treated at a single institution. Fourth, the retrospective design precluded any analysis of patients who were not considered eligible for postoperative therapy because of their performance status.

In conclusion, we retrospectively evaluated patients with pN+ ESCC who underwent surgery followed by adjuvant chemotherapy or chemoradiotherapy. The results indicate that S + CRT was associated with a substantial survival advantage in this setting. Therefore, further work is needed to better understand the role of adjuvant chemoradiotherapy for patients who have undergone esophagectomy for pN+ ESCC.

## Supplementary information

**Additional file 1. **Supporting Table 1: univariate analysis of factors influencing overall survival and disease-free survival in the matched groups. Supporting Figure 1A–D: For patients with KPS scores of 90–100, the S + CT and S + CRT groups had similar 5-year rates of OS and DFS. For patients with KPS scores of 70–80, the S + CRT group had significantly better 5-year rates of OS (39.1% vs. 17.8%, *p* = 0.011) and DFS (31.9% vs. 13.7%, *p* = 0.038). DFS: disease-free survival; OS: overall survival; S + CT: postoperative chemotherapy; S + CRT: postoperative chemoradiotherapy. Supporting Figure 2A–D: For patients with pT1–2 status, the S + CT and S + CRT groups had similar 5-year rates of OS and DFS. For patients with pT3–4 status, the S + CRT group had significantly better 5-year rates of OS (39.6% vs. 23.9%, *p* = 0.043) and DFS (35.2% vs. 23.2%, *p* = 0.016). DFS: disease-free survival; OS: overall survival; S + CT: postoperative chemotherapy; S + CRT: postoperative chemoradiotherapy. Supporting Figure 3A–D: For patients with pN1 status, the S + CT and S + CRT group had similar 5-year rates of OS and DFS. For patients with pN2–3 status, the S + CRT group had significantly better 5-year rates of OS (57.3% vs. 36.4%, *p* = 0.007) and DFS (52.1% vs. 36.5%, *p* = 0.007). DFS: disease-free survival; OS: overall survival; S + CT: postoperative chemotherapy; S + CRT: postoperative chemoradiotherapy.

## Data Availability

The data supporting the findings of this study are available from the corresponding author upon reasonable request.

## Abbreviations

ESCC: Esophageal Squamous Cell Carcinoma
S + CT: Esophagectomy and adjuvant chemotherapy
S + CRT: Esophagectomy and adjuvant chemoradiotherapy
OS: Overall survival
DFS: Disease-free survival
NCRT: Neoadjuvant chemoradiotherapy
pN +: Pathologic lymph node metastasis.
LRR: Local-regional recurrence
HRs: Hazard ratios
CIs: Confidence intervals
S1: S1 stand for Gimeracil and Oteracil Porassium Capsules
MIE: Minimally invasive esophagectomy
