# Supplementary material for: Postoperative adjuvant chemotherapy versus chemoradiotherapy for node-positive esophageal squamous cell carcinoma: a propensity score-matched analysis
Source: Radiat Oncol. 2020 May 24;15:119. doi: 10.1186/s13014-020-01557-9 (PMC7245784; doi:10.1186/s13014-020-01557-9)
Supplement: Supplementary file 1 — Additional file 1. Supporting Table 1: univariate analysis of factors influencing overall survival and disease-free survival in the matched groups. Supporting Figure 1A–D: For patients with KPS scores of 90–100, the S + CT and S + CRT groups had similar 5-year rates of OS and DFS. For patients with KPS scores of 70–80, the S + CRT group had significantly better 5-year rates of OS (39.1% vs. 17.8%, p = 0.011) and DFS (31.9% vs. 13.7%, p = 0.038). DFS: disease-free survival; OS: overall survival; S + CT: postoperative chemotherapy; S + CRT: postoperative chemoradiotherapy. Supporting Figure 2A–D: For patients with pT1–2 status, the S + CT and S + CRT groups had similar 5-year rates of OS and DFS. For patients with pT3–4 status, the S + CRT group had significantly better 5-year rates of OS (39.6% vs. 23.9%, p = 0.043) and DFS (35.2% vs. 23.2%, p = 0.016). DFS: disease-free survival; OS: overall survival; S + CT: postoperative chemotherapy; S + CRT: postoperative chemoradiotherapy. Supporting Figure 3A–D: For patients with pN1 status, the S + CT and S + CRT group had similar 5-year rates of OS and DFS. For patients with pN2–3 status, the S + CRT group had significantly better 5-year rates of OS (57.3% vs. 36.4%, p = 0.007) and DFS (52.1% vs. 36.5%, p = 0.007). DFS: disease-free survival; OS: overall survival; S + CT: postoperative chemotherapy; S + CRT: postoperative chemoradiotherapy. [file 13014_2020_1557_MOESM1_ESM.doc]

| **Support Table 1: univariate analysis of factors influencing overall survival and disease-free survival in the matched groups** | | | | | | |
| --- | --- | --- | --- | --- | --- | --- |
| Factors | level | Overall Survival |  |  | Disease-free Survival |  |
|  |  | HR | Pvalue |  | HR | Pvalue |
| Age | ≤65 | 1 |  |  | 1 |  |
|  | >65 | 1.08(0.83-1.42) | 0.558 |  | 1.01(0.78-1.31) | 0.931 |
| Sex | Male | 1 |  |  | 1 |  |
|  | Female | 0.84(0.61-1.16) | 0.291 |  | 0.76(0.56-1.05) | 0.092 |
| KPS | 90-100 | 1 |  |  | 1 |  |
|  | 70-80 | 1.43(1.13-1.8) | 0.002 |  | 1.46(1.17-1.82) | 0.001 |
| Operation type | Open | 1 |  |  | 1 |  |
|  | mini | 0.77(0.6-0.98) | 0.031 |  | 0.76(0.61-0.96) | 0.019 |
| Esophagutomy: | two fields | 1 |  |  | 1 |  |
|  | three fields | 1.14(0.88-1.47) | 0.324 |  | 1.16(0.91-1.48) | 0.23 |
| LengthGroup: | ≤5cm | 1 |  |  | 1 |  |
|  | >5cm | 1.01(0.76-1.33) | 0.954 |  | 1(0.77-1.3) | 0.99 |
| Pathological differentiation | Well (G1) | 1 |  |  | 1 |  |
|  | Moderate (G2) | 1.1(0.78-1.56) | 0.594 |  | 1.17(0.83-1.64) | 0.364 |
|  | Poor or undifferentiated (G3–4) | 1.07(0.75-1.51) | 0.708 |  | 1.14(0.81-1.6) | 0.453 |
| Location | Upper site | 1 |  |  | 1 |  |
|  | Middle site | 0.95(0.73-1.24) | 0.709 |  | 1.06(0.82-1.37) | 0.658 |
|  | Lower site | 1.03(0.74-1.44) | 0.865 |  | 1.17(0.85-1.61) | 0.35 |
| Margin: | R0 | 1 |  |  | 1 |  |
|  | R1-2 | 1.7(1.21-2.4) | 0.002 |  | 1.48(1.05-2.08) | 0.025 |
| VarscularInvasion: | No | 1 |  |  | 1 |  |
|  | Yes | 1.51(1.16-1.97) | 0.002 |  | 1.39(1.08-1.78) | 0.011 |
| NeuroInvasion: | No | 1 |  |  | 1 |  |
|  | Yes | 1.28(0.98-1.67) | 0.074 |  | 1.38(1.07-1.77) | 0.013 |
| NodeGroup: | ≥20 | 1 |  |  | 1 |  |
|  | ＜20 | 0.86(0.68-1.08) | 0.201 |  | 0.81(0.65-1.01) | 0.063 |
| PathTstage | T1 | 1 |  |  | 1 |  |
|  | T2 | 2.69(0.95-7.58) | 0.061 |  | 1.7(0.76-3.79) | 0.197 |
|  | T3 | 3.36(1.25-9.07) | 0.017 |  | 2.16(1.01-4.58) | 0.046 |
|  | T4a | 6.09(2.2-16.86) | 0.001 |  | 3.54(1.61-7.78) | 0.002 |
|  | T4b | 20.74(5.82-73.93) | <0.001 |  | 10.64(3.55-31.92) | <0.001 |
| PathNstage | N1 | 1 |  |  | 1 |  |
| PathNstage:3 | N2 | 2.11(1.64-2.72) | <0.001 |  | 1.93(1.51-2.45) | <0.001 |
|  | N3 | 3.06(2.18-4.29) | <0.001 |  | 2.68(1.94-3.72) | <0.001 |
| Path8thstage | IIB | 1 |  |  | 1 |  |
|  | IIIA | 3.5(0.82-14.99) | 0.092 |  | 1.66(0.64-4.36) | 0.299 |
|  | IIIB | 5.43(1.34-21.89) | 0.018 |  | 2.32(0.96-5.65) | 0.063 |
|  | IVA | 14.57(3.56-59.55) | <0.001 |  | 5.44(2.19-13.48) | <0.001 |
| AdjuvantTherapy: | S+CT | 1 |  |  | 1 |  |
|  | S+CRT | 0.77(0.61-0.97) | 0.029 |  | 0.75(0.6-0.94) | 0.012 |

Note: AJCC: American Joint Committee on Cancer, S: surgery alone, S+CT: postoperative chemotherapy, S+CRT: postoperative chemoradiotherapy, OS, overall survival; DFS, disease-free survival; HR, hazard ratio; CI, confident interval. KPS: Karnofsky Performance Status.


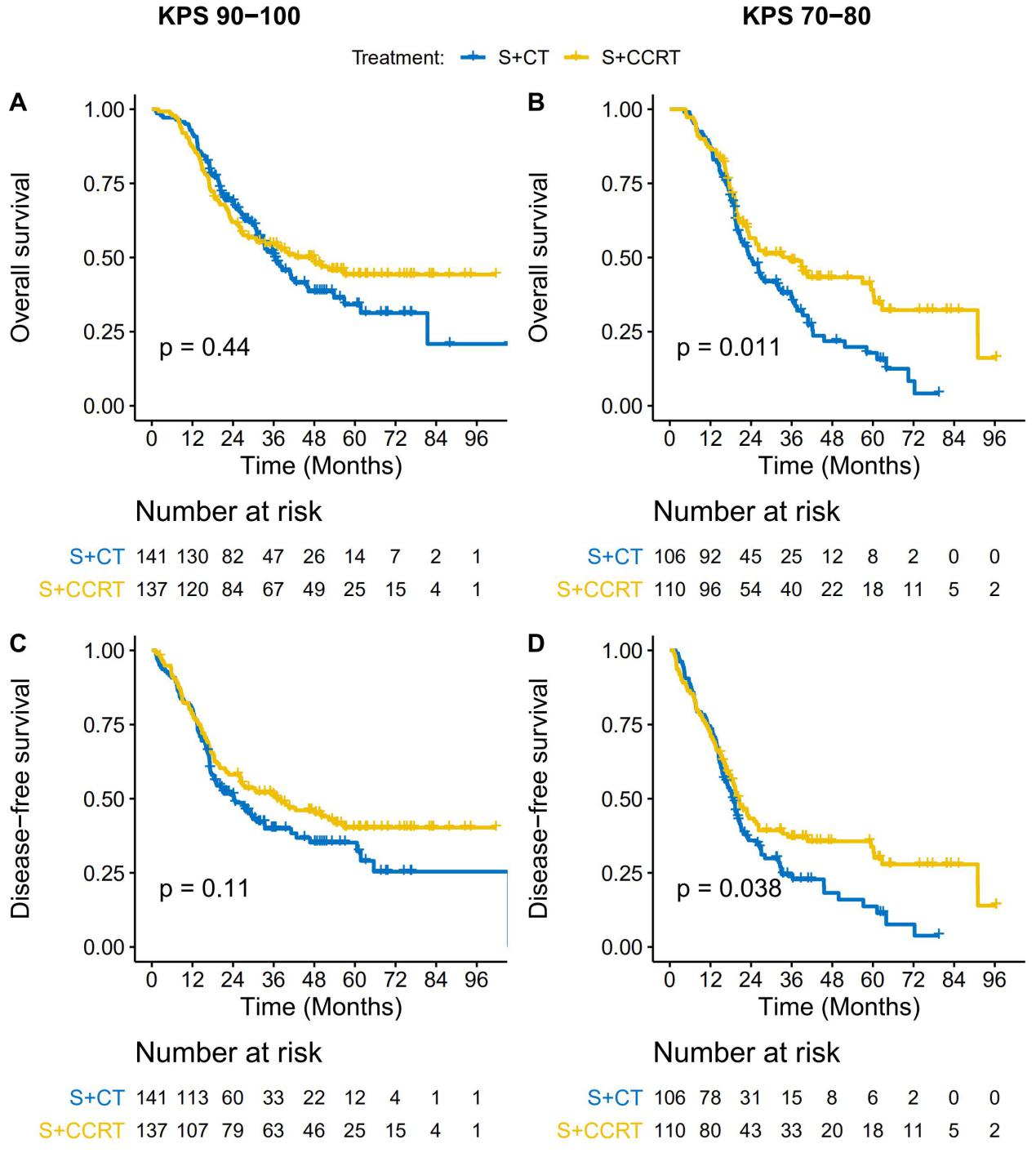
Support Figure 1A-D: For patients with KPS scores of 90–100, the S+CRT group had similar 5-year rates of OS and DFS, relative to the S+CT group. For patient with KPS scores of 70–80, the S+CRT group had significantly better 5-year rates of OS (39.1% vs. 17.8%, p=0.011) and DFS (31.9% vs. 13.7%, p=0.038).


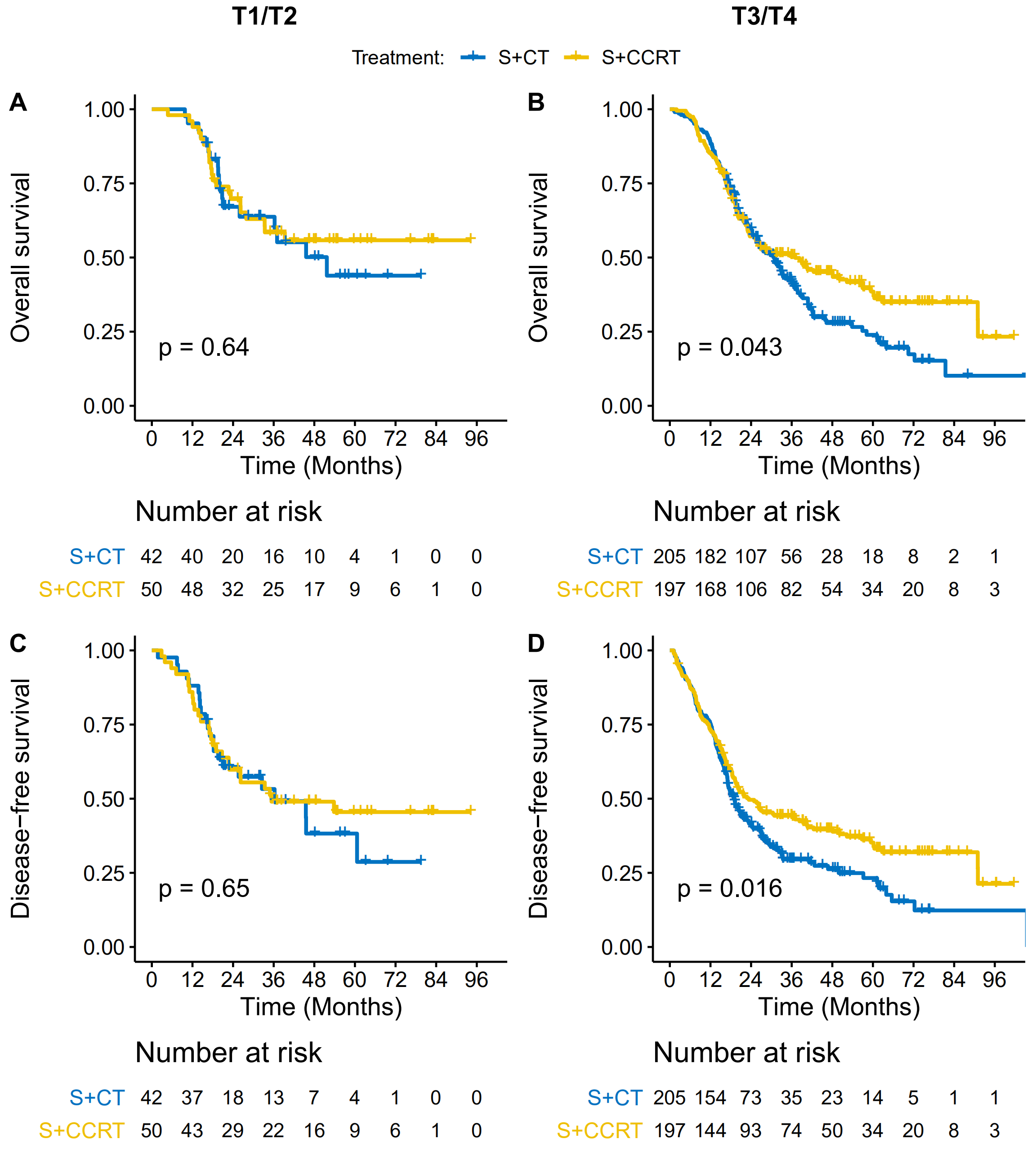


Support Figure 2A-D: For patients with pT1–2 status, the S+CRT group had similar 5-year rates of OS and DFS, relative to the S+CT group. For patients with pT3–4 status, the S+CRT group had significantly better 5-year rates of OS (39.6% vs. 23.9%, p=0.043) and DFS (35.2% vs. 23.2%, p=0.016).


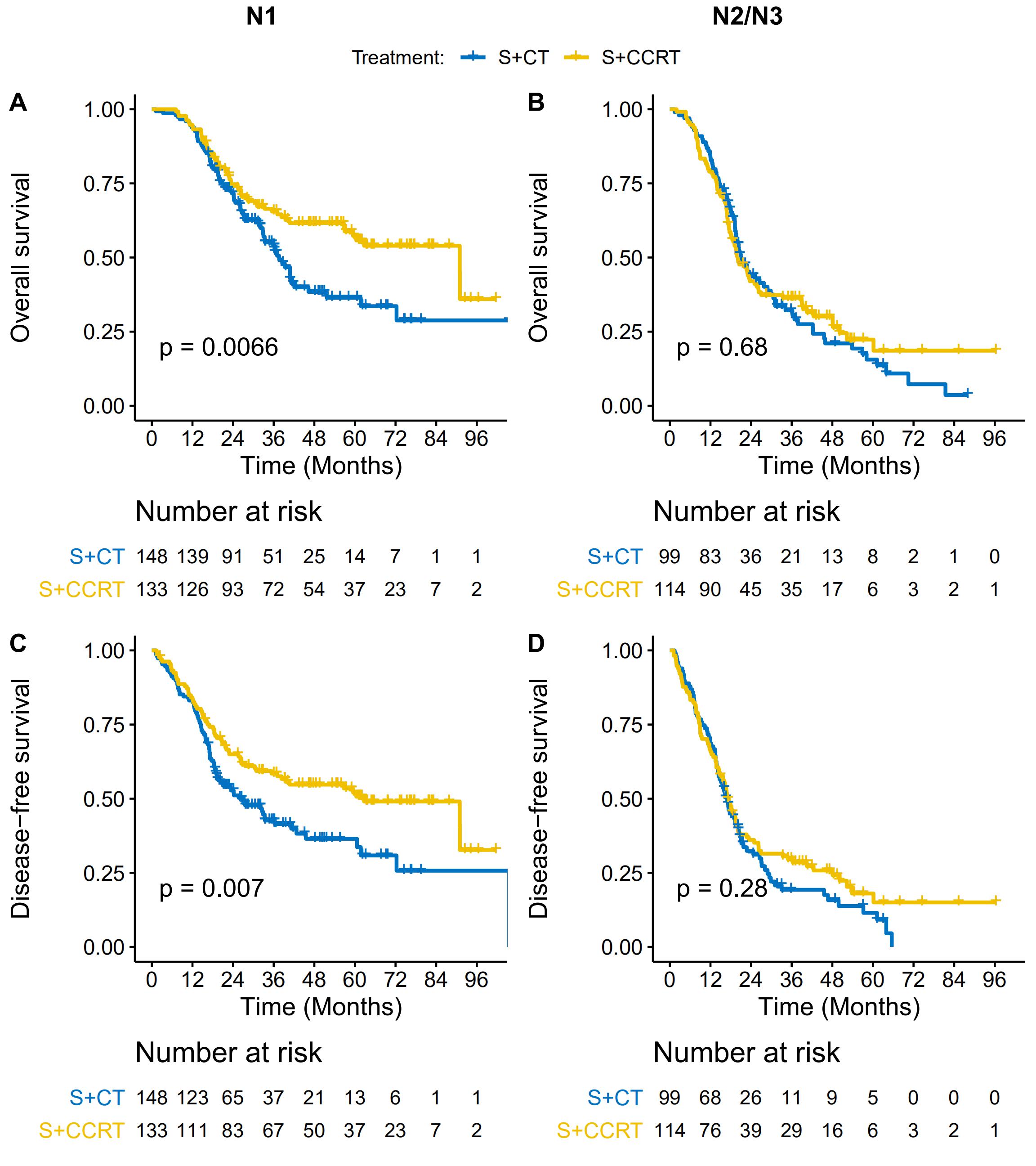


Support figure 3A-D:For patients with pN1 status, the S+CRT group had similar 5-year rates of OS and DFS, relative to the S+CT group. For patient with pN2–3 status, the S+CRT group had significantly better 5-year rates of OS (57.3% vs. 36.4%, p=0.007) and DFS (52.1% vs. 36.5%, p=0.007).
